# Network Biology: Current Status, Applications, Challenges, and Future Prospects

**DOI:** 10.1002/mco2.71065

**Published:** 2026-10-13

**Authors:** Xinmeng Li, Laizhong Zhang, Yawen Ding, Kai Li, Shiyuan Chang, Linsen Pei, Zhengwei Li, Zhenzhen Chen

**Affiliations:** ^1^ Aging Decoding and Regeneration Institute, School of Life Sciences Zhengzhou University Zhengzhou China; ^2^ The First Affiliated Hospital of Henan University Kaifeng China; ^3^ NHC Key Laboratory of Prevention and Treatment of Central Asia High Incidence Diseases, Key Laboratory of Xinjiang Endemic and Ethnic Diseases Shihezi University School of Medicine Shihezi China

**Keywords:** interorgan interaction networks, microbial networks, molecular interaction networks, network biology, single‐cell networks

## Abstract

Network biology provides a systems‐level framework for understanding how interactions among molecular components, cells, microbial communities, tissues, and organs generate biological function and disease phenotypes. Recent advances in multiomics, single‐cell and spatial profiling, network inference, organoid and organ‐on‐chip technologies, computational modeling, and artificial intelligence have shifted the field from static molecular maps toward dynamic, multiscale, and predictive network models. Major branches include molecular interaction networks, intracellular regulatory networks, single‐cell and multicellular networks, microbial networks, disease networks, and interorgan interaction networks. Experimental and computational approaches are compared with respect to network reconstruction, causal validation, and cross‐scale integration. Biomedical applications span disease classification, biomarker discovery, therapeutic‐target identification, drug–response prediction, regenerative medicine, and precision healthcare. Particular emphasis is placed on interorgan interaction networks, in which carrier, route, range, timescale, recipient specificity, directionality, and feedback are treated as distinct mechanistic dimensions while mechanistic confidence is distinguished from translational maturity. Persistent challenges include incomplete context‐specific network reconstruction, uncertain causal directionality, limited cross‐scale integration, model interpretability, and insufficient clinical validation. Further progress will depend on integrating longitudinal and perturbation‐resolved measurements with human‐relevant experimental systems and multiscale computational models that quantify uncertainty and support prospective validation of patient‐specific predictions.

## Graphical abstract

Network biology integrates molecular interaction, intracellular regulatory, single‐cell and multicellular, microbial and host–microbiome, disease, and interorgan networks across biological scales. Perturbation models, multiomics, spatial profiling, computational inference, mechanistic modeling, and AI enable dynamic network reconstruction and causal validation, supporting biomarker discovery, therapeutic‐target identification, drug–response prediction, regenerative medicine, and precision healthcare.

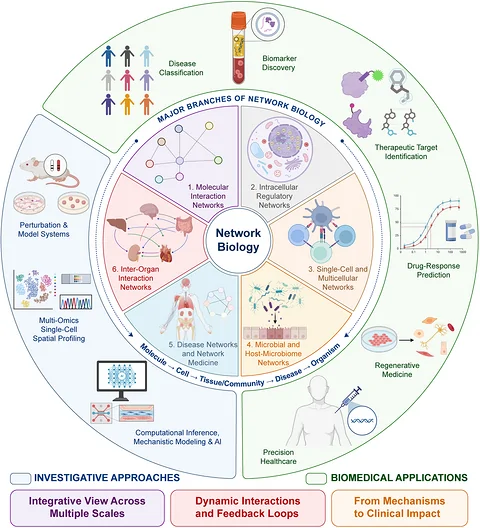

## 1 Introduction

Modern biomedical research now generates rapidly expanding, high‐dimensional molecular, cellular, imaging, and physiological datasets, yet translating these data‐rich discoveries into clinically validated interventions for complex chronic diseases remains challenging [1, 2]. One reason is that disorders such as heart failure, metabolic syndrome, Type 2 diabetes, and neurodegeneration rarely arise from isolated defects in a single gene, protein, cell type, or organ. Instead, they reflect coordinated disturbances across molecular pathways, cellular states, tissue microenvironments, microbial communities, and systemic physiology [3, 4]. Reductionist approaches remain indispensable for defining individual components and mechanisms, but they often cannot fully explain emergent properties such as robustness, redundancy, compensatory adaptation, and nonlinear feedback [5, 6]. These properties determine whether a local perturbation is buffered, remains confined, or propagates across biological scales to produce multiorgan dysfunction [7, 8]. Accordingly, a systems‐level framework is needed to integrate heterogeneous evidence and explain how coordinated interactions maintain health, shape disease susceptibility, and influence therapeutic response.

Network biology provides such a framework by representing genes, proteins, metabolites, regulatory elements, cells, microorganisms, tissues, and organs as nodes linked by physical, regulatory, metabolic, ecological, or informational relationships [9, 10]. By combining network topology with dynamic modeling, perturbation experiments, and context‐specific data, it seeks to identify functional modules and determine how their organization produces adaptation or disease [11]. Early work centered on protein–protein interaction, gene‐regulatory, signaling, and metabolic networks, revealing recurrent organizational features including hubs, bottlenecks, motifs, and communities [12]. Single‐cell and spatial profiling now resolve cell‐state‐specific regulatory programs and multicellular communication systems that are obscured in bulk tissues [13, 14]. Metagenomic, metatranscriptomic, and metabolomic approaches have similarly extended network analysis to microbial communities and host–microbiome interactions [15]. Disease‐network analysis connects molecular modules with comorbidity, therapeutic response, and drug repositioning, whereas interorgan interaction networks extend this logic to organismal physiology [16]. At the organismal scale, signals can propagate between organs through circulating mediators, vesicles or cells, and through anatomically connected neural pathways. Mechanical conditions can modify these interactions and their downstream effects [17, 18]. However, major branches of network biology are often investigated separately, and the mechanisms by which perturbations propagate across molecular, cellular, tissue, and organismal scales remain incompletely understood [19].

Technological advances are accelerating the transition from static, descriptive maps to context‐specific, dynamic, and experimentally testable network models [20]. Physical‐interaction mapping, proximity labeling, CRISPR screening, Perturb‐seq, single‐cell transcriptomics and epigenomics, spatial profiling, longitudinal multiomics, isotope tracing, and organ‐to‐organ protein‐trafficking assays provide complementary information about network structure, cellular context, temporal order, and signal transport [21, 22]. Organoids, assembloids, animal models, microfluidic organ‐on‐chip platforms, and engineered or bioprinted tissues extend perturbation analysis to multicellular, tissue, and organ scales [23, 24]. Cross‐organ imaging genetics and integrated multiomics can further reveal coordinated phenotypes that are not apparent from single‐organ measurements [25]. In parallel, network propagation, causal graphical models, mechanistic simulation, artificial intelligence, and multiorgan digital twins provide increasingly powerful tools for integrating heterogeneous data and predicting system‐level responses [26]. Despite this progress, interaction maps remain incomplete and context dependent, inferred edges are often associative rather than causal, and temporal sampling is usually insufficient to resolve directionality. Data sparsity, batch effects, compositional bias, parameter nonidentifiability, limited interpretability, sex‐ and age‐dependent variation, and imperfect transferability from preclinical models to humans further restrict reproducibility and generalizability [27]. Microphysiological systems face additional challenges involving quantitative standardization, metadata quality, interlaboratory reproducibility, scalability, and regulatory acceptance [28, 29]. Addressing these limitations requires not only improved technologies but also a coherent multiscale framework that connects network structure, causal perturbation, physiological function, and clinical outcome [30]. Recent reviews have addressed domains of interorgan communication, but an integrated multiscale framework linking these fields to causal validation and clinical translation remains lacking [31, 32].

Accordingly, the scope spans molecular interaction networks, intracellular regulatory networks, single‐cell and multicellular networks, microbial and host–microbiome networks, disease networks, and interorgan interaction networks. Experimental, perturbational, multiomics, spatial, and computational approaches are compared with respect to network reconstruction and validation, followed by applications in disease classification, biomarker discovery, therapeutic‐target prioritization, drug–response prediction, regenerative medicine, and precision healthcare. Persistent challenges involving data heterogeneity, network dynamics, causal inference, cross‐scale integration, model interpretability, standardization, validation, and clinical governance are considered in relation to future prospects. Particular emphasis is placed on interorgan interaction networks as an organism‐level extension of network biology. Figure 1 presents the resulting multiscale framework by linking the six major branches with representative investigative approaches and biomedical applications.

**FIGURE 1:**
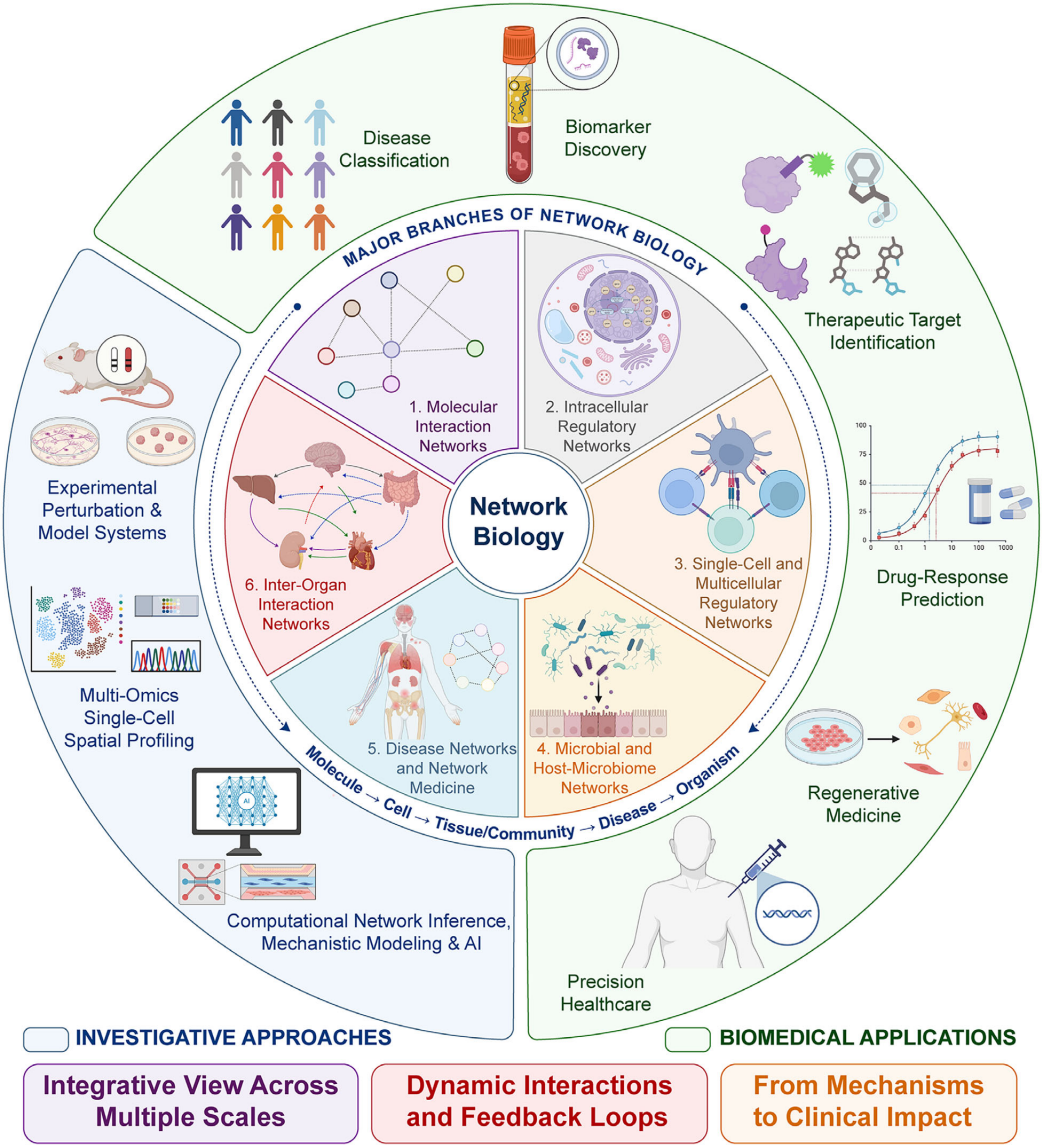
Network biology across biological scales, investigative approaches, and biomedical applications. Six branches connect molecular and cellular interactions with tissue, microbial, disease, and interorgan networks. Experimental perturbation, molecular profiling, and computational analysis link network reconstruction to mechanistic testing and translational research. Curves and network edges are schematic. Created with BioRender.com.

## 2 Current Status and Major Branches of Network Biology

Network biology spans molecular, intracellular, multicellular, microbial, disease, and interorgan scales. Although these branches differ in their nodes, edges, data modalities, and characteristic timescales, they share the goal of identifying functional modules and explaining how perturbations propagate to phenotype.

### 2.1 Molecular Interaction Networks

At the molecular scale, network biology represents genes, proteins, noncoding RNAs, metabolites, and other biomolecules as nodes connected by physical, functional, or statistical relationships [33]. Protein–protein interaction networks describe molecular complexes and signaling assemblies, whereas gene coexpression and regulatory networks capture coordinated transcriptional programs [34]. Metabolic networks represent biochemical reactions and flux relationships, and integrated molecular networks combine multiple edge types to identify modules that support specific cellular functions [35]. Network topology can identify highly connected hubs, modular communities, and recurrent motifs that organize molecular connectivity [36]. However, the functional significance of these topological features depends on tissue identity, cellular state, and experimental context.

A major transition in the field has been the shift from static, context‐agnostic reference interactomes toward tissue‐ and cell‐state‐specific networks that capture condition‐dependent interaction dynamics [37]. Tissue‐specific expression, post‐translational modification, subcellular localization, genetic variation, and perturbation responses can substantially rewire molecular connectivity [38]. Current priorities include improving interactome completeness, resolving transient interactions, quantifying edge confidence, and linking network structure to measured function [39].

### 2.2 Intracellular Regulatory Networks

Intracellular regulatory networks coordinate gene expression, signal transduction, metabolism, organelle function, and cell‐fate decisions [10]. Nodes include transcription factors, enhancers, kinases, phosphatases, receptors, metabolites, and regulatory RNAs, whereas their edges denote activation, inhibition, binding, or metabolic conversion. Recurrent regulatory motifs, including feedback and feedforward loops, together with cross‐pathway coupling and partially redundant routes, can confer robust signal processing and adaptation, whereas bistable or history‐dependent regulatory states can generate threshold responses and cellular memory. These properties are central to development, stress responses, immune activation, and drug resistance [11].

Modern reconstruction strategies integrate transcription‐factor motifs, chromatin accessibility, enhancer activity, phosphoproteomics, time‐series measurements, and genetic perturbations [40]. Tissue‐specific regulatory circuit maps further show that disease‐associated variants often converge within regulatory modules active in disease‐relevant cell types or tissues. However, regulatory edges inferred from correlation or motif enrichment remain hypotheses until supported by perturbation, temporal precedence, dose–response relationships, or rescue experiments [41].

### 2.3 Single‐Cell and Multicellular Regulatory Networks

Single‐cell network biology resolves regulatory relationships obscured in bulk tissues by integrating single‐cell transcriptomic, epigenomic, proteomic, and spatial data to reconstruct cell‐state‐specific regulatory programs and gene‐regulatory networks [42]. These multimodal datasets also enable the inference of developmental trajectories and spatially organized multicellular communication networks [43]. Methods such as SCENIC and SCENIC+ infer transcription‐factor‐centered regulons and enhancer‐driven regulatory programs [13, 14], whereas ligand–receptor frameworks such as LIANA+ estimate communication among defined cell populations [44].

These approaches have transformed studies of development, tissue homeostasis, immune organization, and the tumor microenvironment [45, 46]. In a mouse model, FGF21 overexpression in medullary thymic epithelial cells altered mTOR signaling in cortical thymic epithelial cells and supported thymus maintenance, providing an example of local multicellular regulation within a single organ [47]. Nevertheless, sparse measurements, batch effects, uncertain ligand–receptor activity, and incomplete spatial information can generate false‐positive or noncausal edges. Computationally inferred communication should therefore be treated as a set of testable hypotheses and evaluated using orthogonal spatial measurements, ligand‐ or receptor‐directed perturbations, downstream signaling readouts, and functional validation [48].

### 2.4 Microbial and Host–Microbiome Networks

Microbial network biology investigates interactions among microorganisms and between microbial communities and their hosts. Microbial taxa, genes, pathways, phages, and metabolites can function as nodes, whereas competition, cooperation, cross‐feeding, predation, and environmental filtering define different edge types. Metagenomic, metatranscriptomic, metabolomic, and longitudinal abundance data can be integrated to identify modules and candidate keystone species, while time‐series models can test how perturbations propagate through ecological networks [49].

Because microbiome abundance data are compositional, co‐occurrence does not necessarily indicate a direct ecological interaction, and apparent associations may reflect indirect covariance or unmeasured contextual factors [50]. Controlled metabolite perturbation and longitudinal dynamic modeling can help distinguish direct ecological interactions from associations generated by shared environmental or host factors [51, 52]. Host–microbiome networks connect microbial ecology with immune, metabolic, neural, and interorgan regulation and thus form a major bridge between community‐level and organism‐level network biology [53].

### 2.5 Disease Networks and Network Medicine

Disease‐network analysis considers complex disorders as perturbations of interconnected molecular and cellular modules (CMs) rather than as consequences of isolated genes. Disease‐associated genes often localize within specific interactome neighborhoods, and the proximity or overlap of these modules can help explain shared mechanisms and comorbidity. Network propagation and disease‐module detection can prioritize candidate disease genes and expand incomplete disease modules [54].

Network medicine can support drug repurposing, rational combination therapy, and designed polypharmacology by mapping drug targets onto disease‐relevant modules and evaluating how single‐ or multitarget interventions perturb functionally connected network components rather than isolated proteins [55]. The emerging goal is to construct patient‐specific networks that integrate genetic variation, molecular profiles, cellular composition, environmental exposure, and clinical trajectories [56]. Translation requires robust benchmarking, prospective validation, and clear evidence that network‐based predictions add value beyond established clinical models [57]. Table 1 integrates the six major network branches with their principal experimental and computational approaches, applications, and limitations.

**TABLE 1:** Network branches and investigative approaches.

Network branch	Nodes	Edges	Experimental approach	Computational approach	Application	Strength	Limitation	Validation	Refs.
Molecular	Proteins, RNAs, metabolites	Physical or functional links	AP‐MS, proximity labeling, molecular profiling	Interaction and metabolic models	Target discovery	Pathway mapping	Incomplete context‐specific maps	Orthogonal binding tests	[9, 12, 34, 37]
Intracellular	TFs, enhancers, genes, kinases	Activation or inhibition	CRISPR, Perturb‐seq, regulatory profiling	Dynamic regulatory models	Cell‐state control	Perturbation‐response mapping	Model identifiability	Causal perturbation controls	[10, 11, 58]
Single‐cell and multicellular	Cell states, regulons, ligands, receptors	Regulation or local signaling	Single‐cell omics, spatial omics, coculture perturbation	SCENIC, SCENIC+, LIANA+	Tissue organization	Cell‐specific interaction mapping	Sparsity and batch effects	Ligand–receptor perturbation	[13, 14, 42, 44, 48, 59]
Microbial	Taxa, genes, pathways, metabolites	Ecological or metabolic links	Metagenomics, metabolomics, community perturbation	Time‐series ecological models	Community dynamics	Host–microbiome mapping	Compositional bias	Controlled interaction tests	[15, 49, 50, 51, 52, 60]
Disease	Disease genes, modules, drug targets	Shared or perturbed functions	Single‐cell disease profiling	Propagation, proximity, graph learning	Target prioritization	Disease‐module mapping	Incomplete interactomes	Independent clinical benchmarks	[5, 6, 54, 55, 61, 62]
Interorgan	Source and recipient organs or cells	Cross‐organ signal transfer	Tracing, parabiosis, multiorgan chips, longitudinal omics	Mechanistic models, AI, digital twins	Mechanism reconstruction	Cross‐organ response mapping	Scaling and species differences	Tracing and functional rescue	[17, 22, 24, 26, 39, 63, 64, 65, 66, 67, 68, 69, 70, 71, 72

### 2.6 Interorgan Interaction Networks

Interorgan interaction networks represent the organism‐level extension of network biology, in which perturbations originating in one tissue are transmitted through defined routes and reorganize physiological states in anatomically distant organs [73, 74]. Unlike molecular or intracellular edges, which commonly denote binding, regulation, or metabolic conversion, interorgan edges may combine the transfer of soluble mediators, EVs or migrating cells with transmission through neural circuits [17, 67, 68]. Operationally, an interorgan interaction requires transmission of a signal or signal‐bearing cell from a source tissue through an identifiable vascular, lymphatic, neural, or other physiologically defined route, together with a measurable response in an anatomically distinct recipient organ [75]. Local cell–cell communication, interactions between regions of the same organ, and tumor‐microenvironment signaling without an extra‐organ route are excluded, whereas host–microbiome interactions are included only when microbial products or microbiota‐induced host responses reach distal organs [60].

Each interorgan edge should therefore be characterized along independent mechanistic dimensions, including signal carrier, transport route, spatial range, response timescale, recipient specificity, directionality, and feedback, rather than assigned to equivalent communication categories. Endocrine and paracrine specify the spatial reach of signaling. EVs identify a physical carrier, whereas neural and immune systems identify participating biological systems whose routes must be specified separately [76]. Mechanical inputs can modify these interactions without necessarily constituting a separate interorgan transmission route [18, 77]. Figure 2 presents five representative cases, one for each subsection below, and Table 2 links mechanistic properties to study‐specific evidence and translational maturity.

**FIGURE 2:**
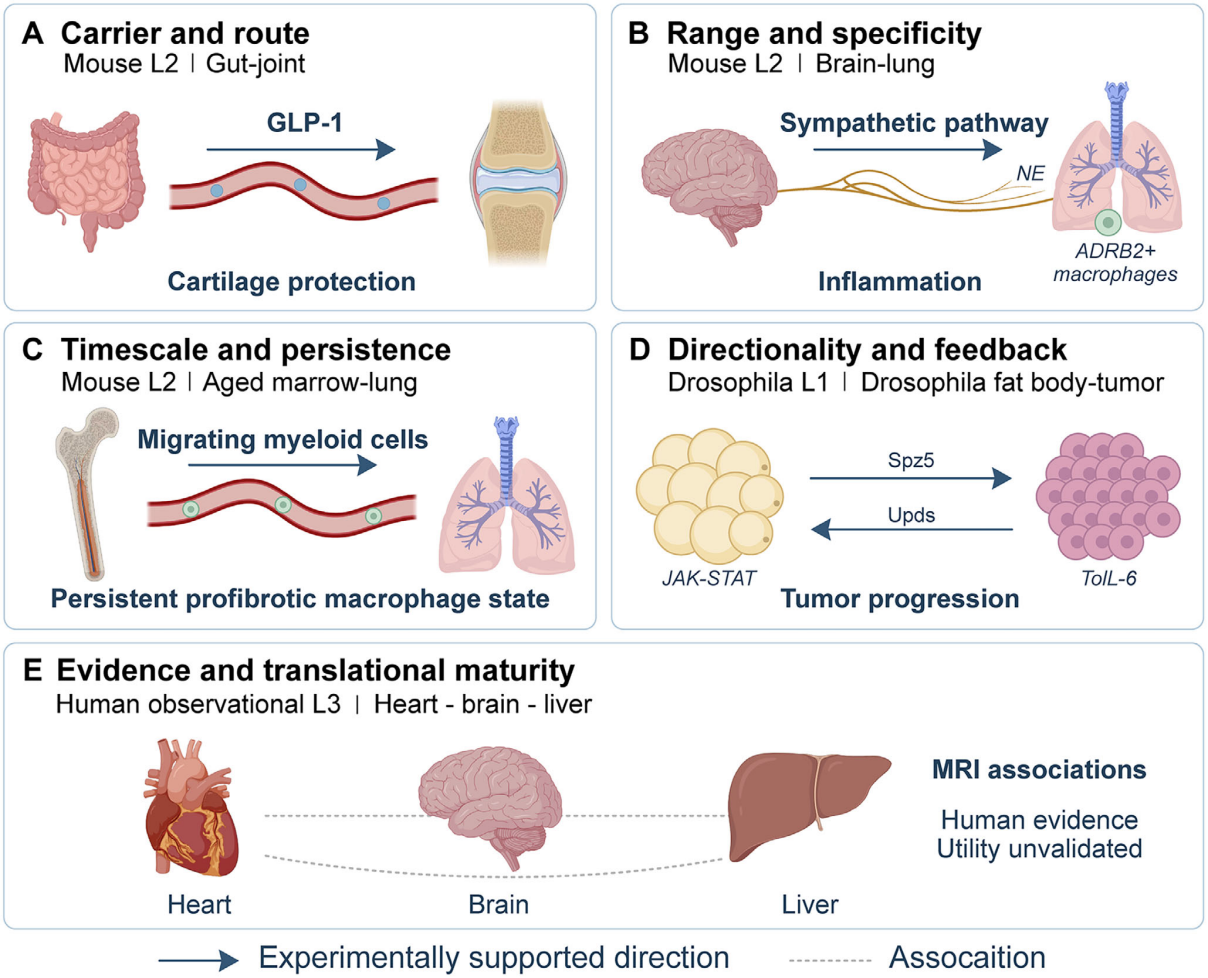
Representative cases illustrating independent mechanistic dimensions and study‐specific evidence in interorgan networks. (A) Intestinal GLP‐1 contributes to cartilage protection in mice, illustrating a soluble mediator and circulatory route. (B) A sympathetic brain‐to‐lung pathway enhances local norepinephrine release onto ADRB2^+^ macrophages, illustrating route and recipient specificity. (C) Aged marrow promotes persistence of profibrotic monocyte‐derived alveolar macrophages after recruitment to the lung. (D) Reciprocal Spz5 and unpaired signaling links the fat body and epithelial tumors in *Drosophila*. (E) Human heart, brain, and liver MRI phenotypes show associations without an established causal direction. Solid arrows denote experimentally supported directions. Undirected dashed lines denote associations. Created with BioRender.com.

**TABLE 2:** Mechanistic dimensions and evidence maturity of representative interorgan axes and experimental platforms.

Example	Carrier or mediator	Route	Range	Recipient	Response timing	Direction	Feedback	Evidence	Level	Implication	Limitation	Refs.
Gut–joint	Soluble GLP‐1	Circulation	Systemic	GLP‐1‐responsive joint tissues	During treatment	Gut to joint	Not established	Mouse receptor perturbations	L2	Cartilage protection	Clinical utility unvalidated	[78]
Brain‐lung	Terminal NE	Sympathetic nerves	Anatomically restricted	ADRB2^+^ macrophages	Acute response	Brain to lung	Not established	Mouse circuit perturbations	L2	Pulmonary inflammation	Human transferability unresolved	[79]
Aged marrow–lung	Migrating myeloid cells	Vascular recruitment	Distant organ	Lung niche	Persistent response	Marrow to lung	Not established	Mouse marrow transplantation	L2	Persistent fibrosis	Transplantation‐model dependence	[80]
Fat body–tumor	Spz5 and Upds	Intertissue secretion	Distant tissues	Tumor and fat body	During tumor progression	Two‐way	Reinforcing loop	Tissue‐specific fly genetics	L1	Tumor progression	Mammalian translation unresolved	[81]
Heart‐brain‐liver	Unresolved	Unresolved	Unresolved	No directed recipient assigned	Unresolved	Unresolved	Unresolved	Human MRI associations	L3	Candidate imaging relationships	Clinical utility unvalidated	[82]
Aged marrow to distant tissues	EVs carrying microRNAs	Systemic circulation	Multiple tissues	PPARα‐responsive tissues	Study‐specific	Marrow to tissues	Not established	Mouse interventions	L2	Senescence‐associated phenotypes	Human EV transport unproven	[70, 83]
Heart‐liver‐bone‐skin chip	Drugs and metabolites	Vascular perfusion	Within‐chip	Engineered tissue niches	During dosing	Perfusate to tissues	No whole‐body loop	Human tissue chip	L1	Metabolism‐toxicity coupling	Personalized utility unvalidated	[71]
Gut–liver‐adipose chip	Perfusate‐borne tissue products	Microfluidic connections	Within‐chip	Gut, liver, adipose modules	During culture	Route dependent	Unresolved	In vitro disease model	L1	Metabolic interaction modeling	Patient‐specific validation lacking	[72]

Carrier or mediator identifies the physical vehicle or signaling agent and is distinct from its route. Response timing describes the reported recipient response or observation window, not carrier transit. Treatment and culture windows should not be interpreted as intrinsic response kinetics. Level denotes claim‐specific translational maturity. L1, mechanistic or technical proof of concept. L2, disease‐relevant preclinical validation in mammals. L3, observational or exploratory interventional human evidence without established clinical utility. L4, prospective validation for a defined clinical use. The levels do not rank intrinsic study quality.

#### 2.6.1 Signal Carriers and Transport Routes

Soluble hormones, cytokines, and metabolites can enter blood or lymph and act on distant tissues [84, 85, 86, 87]. EVs provide a physical carrier for intercellular cargo [88, 89]. Migrating cells can transfer biological activity through vascular or lymphatic routes, whereas neural pathways transmit activity through connected neurons and release mediators at defined terminals. Local soluble paracrine signaling occurs within a tissue microenvironment and does not itself constitute an interorgan edge, although it can initiate a systemic relay [90]. Systemic EV transport must therefore be assessed separately from local soluble signaling [70].

The gut–joint axis provides a representative example of a soluble signal and its transport route. Microbiota‐dependent glycoursodeoxycholic acid (GUDCA) metabolism regulates intestinal farnesoid X receptor (FXR) activity and glucagon‐like peptide 1 (GLP‐1) secretion. In mice, FXR suppression alleviates osteoarthritis, GLP‐1 receptor blockade attenuates this effect, and receptor activation mitigates disease, collectively supporting a mechanistically defined gut–joint relay, although the corresponding human evidence remains observational (Figure 2A) [78]. During acute kidney injury, renal tubular cells release osteopontin (OPN) into the circulation, contributing to pulmonary endothelial permeability, inflammation, and respiratory failure in mice [69].

Microbial metabolites constitute an additional class of soluble carriers, as illustrated by exercise‐associated microbiota‐derived formate, which enhances tumor‐specific CD8^+^ T‐cell activity and immunotherapy responses, and microbiota‐dependent phenylacetic acid, which promotes endothelial senescence during aging [91, 92]. Such metabolite‐mediated systemic effects may operate through mechanisms that differ from classical hormone‐receptor signaling and should therefore be evaluated according to their specific source, route, and recipient responses. Muscle‐derived L‐BAIBA provides a further example of a soluble signal affecting extracellular‐matrix homeostasis in the intervertebral disk [93].

In aging mice, bone marrow monocyte/macrophage‐derived EVs were reported to propagate senescence‐associated phenotypes through microRNA‐dependent suppression of PPARα, with fenofibrate improving selected outcomes [83], although the reported human associations with fenofibrate do not establish EV‐mediated transport in patients. Carbon monoxide‐induced autophagy in administered human mesenchymal stromal cells can also alter EV cargo and improve outcomes in septic mice, but this is an experimental cell‐therapy model rather than proof of an endogenous organ‐to‐organ source [94]. Across proposed EV axes, source attribution, biodistribution, recipient‐cell uptake, cargo‐dependent activity, and rescue require separate assessment [95, 96].

Immune pathways likewise combine cell migration, soluble signaling, and shared vascular or lymphatic routes [97, 98]. Gut‐derived immune responses can alter pulmonary immunity or brain‐resident T‐cell states in experimental models [99, 100]. Dendritic cells from the pancreas, liver, and duodenum can converge in shared lymph nodes, establishing immune crosstalk without implying direct migration from one organ into another [101]. Gut–skin interactions further illustrate that changes in inflammation or microbial composition require route‐specific testing before a directed interorgan mechanism is assigned [102].

#### 2.6.2 Spatial Range and Recipient Specificity

Systemic exposure does not necessarily produce uniform organ responses because soluble ligands and EVs differ in stability, clearance, tissue access, and recipient uptake, while receptor expression and cell state determine whether exposure elicits a functional effect [76, 86, 95]. In contrast, anatomically restricted neural projections provide a distinct basis for target selection, as illustrated by organ‐specific sympathetic neuron subtypes within the celiac‐superior mesenteric ganglion complex [103].

In severe pneumonia models, a central amygdala–rostral ventrolateral medulla (RVLM)‐sympathetic pathway promotes norepinephrine release and activates ADRB2^+^ pulmonary interstitial macrophages, amplifying inflammatory responses and illustrating how anatomical routing and recipient‐cell receptor expression jointly determine neural target specificity (Figure 2B) [79]. Other neural axes couple intestinal L‐cell ADRA2A signaling to GLP‐1 release or intestinal stem‐cell CHRM3‐p38 signaling to stress‐associated regenerative decline [104, 105]. Sleep fragmentation‐associated sympathetic activity can also alter cardiomyocyte copper handling and aggravate ischemia–reperfusion injury [106].

Neural specificity should be evaluated through anatomical tracing, selective activation or inhibition, receptor blockade, and functional rescue, rather than activity mapping alone [107, 108]. Brainstem Dbh‐positive neurons provide a further example of circuit‐specific control of allergen‐induced airway hyperreactivity [109]. Autonomic imbalance can modify the response of recipient organs, underscoring the need to define physiological state as well as circuit anatomy [110].

#### 2.6.3 Response Timescales and Persistence

Signal transmission time and the duration of a recipient response are distinct properties. Neural activity may change rapidly, whereas altered secretion, cell recruitment, immune memory, and tissue remodeling can persist after the initiating signal has subsided [111, 112]. Brain injury‐associated innate immune memory and subsequent cardiac dysfunction illustrate how an initiating insult can produce a delayed systemic effect [113]. Sampling and perturbation should therefore resolve onset, transport, response, and recovery rather than assign a fixed timescale to a communication category.

The aged bone marrow–lung axis provides an example in which recipient‐cell dysfunction persists after hematopoietic‐cell recruitment, as heterochronic marrow transplantation in mice showed that hematopoietic aging increases the accumulation of monocyte‐derived alveolar macrophages with delayed maturation toward a homeostatic phenotype. Reduced pulmonary IL‐10 helps sustain this profibrotic state, whereas regulatory T‐cell‐derived IL‐10 promotes macrophage maturation and attenuates fibrosis (Figure 2C) [80]. The relevant temporal feature is persistent macrophage dysfunction after recruitment, rather than the transit time of circulating cells.

#### 2.6.4 Directionality, Relays, and Feedback

A directed edge, a multiorgan relay, and a reciprocal feedback loop require different evidence. Source‐to‐target activity must be distinguished from parallel responses to a shared driver. In leukemia, CD8^+^ T cells migrate from marrow to intestine and return after immunogenic chemotherapy through stage‐dependent trafficking programs [114]. Opposing migration routes do not by themselves establish a continuously operating feedback loop.

Direct experimental evidence for reciprocal signaling comes from the *Drosophila* fat body–tumor axis, in which fat‐body‐derived Spz5 activates tumor Toll‐6‐dependent Hedgehog signaling, while tumor‐derived unpaired ligands activate fat‐body JAK‐STAT signaling and promote spz5 expression. Tissue‐specific genetic perturbations support this reinforcing circuit and its contribution to tumor progression (Figure 2D) [81]. Feedback should therefore be assigned only when both arms and their functional coupling are supported.

#### 2.6.5 Mechanistic Evidence and Translational Maturity

Mechanistic confidence should be evaluated independently of translational maturity because source‐specific perturbation, route tracing, recipient‐side inhibition, and rescue can establish a causal edge in an experimental model without demonstrating clinical utility [69, 78, 114]. Conversely, cardiac, cerebral, and hepatic MRI phenotypes in 30,444 UK Biobank participants show coordinated human associations without resolving a transport route or causal direction (Figure 2E) [82].

Table 2 integrates carrier, route, range, timescale, recipient specificity, directionality, and feedback with study design and evidence maturity for each selected axis or platform. Panels A–E correspond to the first five rows, followed by EV and experimental‐platform examples. Translational‐maturity levels L1–L4 follow the claim‐specific definitions in Section 4 and do not represent the intrinsic quality of a study or an entire communication system. Mechanistic findings and human associations within the same publication are assessed separately.

Biological networks are organized as interacting layers rather than isolated systems. Molecular interactions shape intracellular states, cellular and microbial networks coordinate tissue functions, and interorgan signals transmit local perturbations to distant organs. Conversely, endocrine, neural, immune, metabolic, and mechanical signals can remodel molecular and cellular programs within recipient tissues. Network biology can therefore be viewed as a network of networks in which biological function emerges from interactions within and across scales [115, 116].

Cross‐scale integration requires explicit consideration of differences in spatial resolution, temporal dynamics, data modalities, and the biological meaning of network edges [116]. A molecular edge may represent physical binding or biochemical conversion, whereas cellular, microbial, and interorgan edges may represent regulation, ecological dependence, signal transport, or physiological coupling. Robust interpretation therefore requires clearly defined assumptions, temporally resolved measurements, targeted perturbation, route tracing, and functional validation rather than simple data concatenation [22].

## 3 Investigative Approaches in Network Biology

Investigative approaches in network biology must capture network structure, dynamics, context dependence, and causal responses across multiple biological scales. Because no single method can reconstruct a complete biological network, physical‐interaction mapping, genetic perturbation, multiomics profiling, single‐cell and spatial measurements, engineered models, and computational inference must be integrated as complementary sources of structural, dynamic, and causal evidence. These approaches span experimental perturbation, multiscale molecular and spatial profiling, and computational modeling, with interorgan platforms providing an organism‐level setting for their integration.

### 3.1 Experimental Perturbation and Model Systems

At molecular and cellular scales, affinity purification‐mass spectrometry, proximity labeling, reporter assays, pooled CRISPR screens, Perturb‐seq, lineage tracing, and targeted pathway perturbations are central to identifying network edges and testing directionality. Perturb‐seq links genetic interventions with single‐cell transcriptional responses, allowing many candidate regulators to be evaluated in parallel and helping distinguish association from functional control. At tissue and organismal scales, organoids, animal models, organ‐on‐chip systems, and multiorgan platforms extend perturbation analysis to multicellular and systemic networks [11, 117].

Deciphering the complex network architectures underlying interorgan interaction depends on the availability of safe, robust, and physiologically relevant experimental models. Recent advances in organoid‐based and multiorgan microphysiological systems, refined in vivo models, and CRISPR‐Cas perturbation technologies have expanded the experimental investigation of interorgan communication [118]. Organoid‐based and microfluidic systems enable controlled interrogation of defined tissue interactions, whereas animal models preserve systemic circulation, neuroendocrine regulation, immune‐cell trafficking, and whole‐body feedback [23, 24]. Tissue‐ or cell‐type‐specific CRISPR perturbations can test the functional necessity of candidate signaling components in vivo, although robust causal validation additionally requires route tracing, target‐organ readouts, and rescue experiments [119]. Building on these approaches, multiorgan‐on‐a‐chip systems integrate multiple organ modules within shared circulatory architectures, enabling dynamic and real‐time analysis of functional coupling [120]. These strategies establish a hierarchical experimental framework spanning levels from the cellular to the organismal scale, supporting mechanistic studies and translational drug discovery.

Organoid platforms range from conventional single‐organ models to vascularized, immunocompetent, and bioprinted organoids, whereas physically connected systems require a defined communication route and functional coupling to qualify as interorgan models [121, 122, 123]. Living‐organ bioprinting and multiscalar circulation frameworks further highlight the importance of hierarchical vascularization, perfusion, and transport for scaling engineered tissues toward organ‐level systems [124, 125]. A platform should be considered interorgan only when it contains at least two anatomically distinct organ‐derived compartments, a defined communication route, and functional readouts attributable to intercompartment signaling, with appropriate physiological scaling and reproducibility. Physiological scaling should additionally consider relative organ or compartment size, medium volume, vascular flow, and exposure conditions to avoid nonphysiological dilution or accumulation of circulating signals. Liver‐islet coculture illustrates functional coupling [126], whereas cerebral and kidney organoid studies highlight model‐specific development, maturation, and standardization issues [127, 128].

Animal models remain essential for preserving systemic physiology and testing the causality of interorgan communication. In cancer‐associated cachexia, tumor‐associated vagal dysregulation depletes hepatic HNF4α, whereas interventions targeting the right cervical vagus nerve attenuate cachexia progression in mice [129]. Cell‐specific glucocorticoid‐receptor knockout models further demonstrated that stress‐induced glucocorticoids promote Th17‐cell differentiation and exacerbate intestinal inflammation [130]. In diabetic kidney disease, gut microbiota‐derived indole‐3‐propionic acid protected glomerular endothelial mitochondria by stabilizing SIRT1, illustrating a microbiota–kidney metabolic axis [131]. Direct corticosterone effects on intestinal stem cells (ISCs) provide a complementary stress‐response mechanism [132]. The examples span neural, endocrine‐immune, and microbial‐metabolic mechanisms for establishing cross‐organ causality, although validation in human‐relevant systems remains necessary. Exogenous FGF1 and FGF4 can enhance skeletal‐muscle glucose disposal through FGFR1c‐AMPK‐GLUT4 signaling, but pharmacological activity does not establish the physiological source and endogenous transport route of an interorgan signal [133, 134]. Regional sympathetic‐sensory nerve interactions likewise remain local unless an extra‐organ route is demonstrated [135].

Establishing causality in interorgan communication requires complementary strategies that identify the signal source, transmission route, recipient tissue, and functional consequence. Parabiosis or cross‐circulation can determine whether an effect is mediated by circulating factors, neural tracing combined with optogenetic or chemogenetic perturbation can resolve circuit‐specific pathways, and isotope tracing can directly track metabolite transfer between tissues [63, 64]. Because no single method establishes the entire causal chain, robust claims should combine route‐specific tracing with source‐ or target‐specific perturbation and phenotypic rescue. Receptor or pathway blockade can test recipient‐side necessity, whereas spatially restricted delivery or locally controlled perturbation can help distinguish route‐specific effects from broader systemic exposure. In multiorgan networks, signaling hierarchy should be interpreted as a context‐dependent sequence of propagation rather than a fixed anatomical ranking. Resolving such a hierarchy requires distinguishing the initiating source organ, the primary recipient organ, secondary downstream organs, and any return signals that close feedback loops.

Future experimental platforms should integrate perfusable vascular networks, tissue‐resident immune compartments, organ‐specific perturbation, high‐fidelity genome editing, and continuous physiological monitoring to improve model fidelity and reproducibility [136]. Combining organoids, in vivo models, and modular multiorgan‐on‐a‐chip systems with longitudinal multiomics may support closed‐loop workflows from in vitro screening to in vivo validation [137]. Figure 3 provides a schematic overview of these integrated experimental platforms.

**FIGURE 3:**
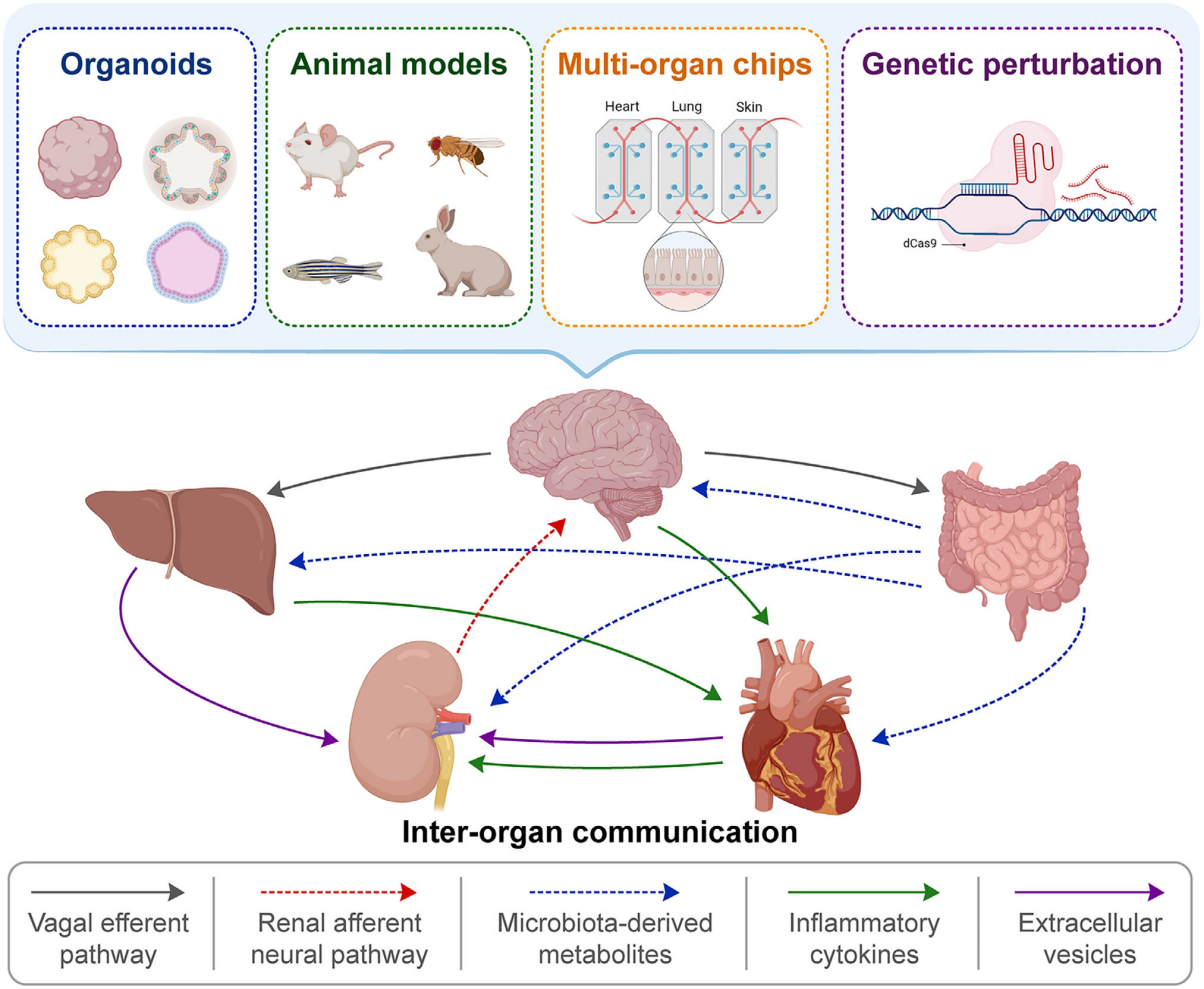
Experimental platforms for testing interorgan communication. Organoids, animal models, multiorgan chips, and genetic perturbation provide complementary control over signal sources, routes, recipients, and functional readouts. The lower network illustrates neural, soluble mediator, and extracellular‐vesicle routes rather than a complete set of validated organ‐to‐organ connections. Afferent neural signaling is distinguished from autonomic efferent output. Created with BioRender.com.

### 3.2 Multiomics, Single‐Cell, and Spatial Profiling

Multiomics and spatial profiling provide the principal observational foundation for reconstructing network biology across molecular, cellular, microbial, tissue, and organ scales. Genomics, transcriptomics, epigenomics, proteomics, metabolomics, and microbiome data identify candidate nodes and edges, whereas single‐cell and spatial technologies resolve cell‐state specificity and local communication [42, 138]. Longitudinal designs establish temporal ordering, whereas controlled perturbations help distinguish upstream regulators from downstream responses. Used together, these approaches strengthen inference of network directionality. In the interorgan context, cross‐tissue profiling can identify the likely sources, transport routes, and recipient‐organ responses of circulating signals. When several organs participate, longitudinal multiorgan sampling can further distinguish an initiating source response from primary recipient‐organ responses, subsequent propagation to secondary organs, and delayed return signals associated with feedback regulation.

Proteomics, in turn, enables the identification of protein mediators involved in interorgan signaling and reveals regulatory patterns at the protein level. For example, lectin affinity chromatography coupled with mass spectrometry has shown that the mannose receptor Mrc1 regulates the abundance of more than 200 mannosylated proteins in the circulation of healthy mice. Loss of Mrc1 leads to aberrant protein accumulation and subsequent multiorgan dysfunction [139]. Using plasma metabolomic data from 274,247 individuals across 107 metabolites, researchers constructed metabolomics‐based biological age models for five organs. Mendelian randomization analyses further supported potential causal relationships between selected MetBAGs and cardiometabolic diseases, revealing distinct interorgan metabolic signatures [140].

Multiomics integration strategies for interorgan studies should be selected according to the biological objective, available data types, and direction of hypothesis generation [33, 138]. Top‐down analyses begin with altered metabolites, proteins, or phenotypes and seek their upstream regulators, whereas bottom‐up analyses trace the consequences of candidate genes or tissue‐specific perturbations across intermediate molecular layers. Network‐based integration complements these approaches by jointly inferring regulatory relationships across multiple omics layers without requiring a strictly predefined hierarchy. Reliable reconstruction of cross‐organ networks further requires spatial or single‐cell resolution, temporal sampling, rigorous control of batch effects, and appropriate tissue deconvolution to distinguish genuine biological signals from technical variation. Ligand–receptor inference can then prioritize candidate communication pathways, although the predicted interactions require perturbation‐based functional validation.

The analytical value of multimodal integration is illustrated by studies combining transcriptomic, single‐cell, proteomic, and computational data to identify osteokines and map bone‐derived signaling toward the brain, liver, and vascular smooth muscle cells [141]. In another example, metatranscriptomics revealed rhythmic expression of a bile salt hydrolase gene in *Dubosiella newyorkensis*, while integration with serum metabolomics linked this microbial activity to glucose homeostasis through the bile acid–pancreatic axis [142]. Such studies illustrate how complementary molecular layers can connect a candidate signal to its cellular source, transport pathway, recipient tissue, and physiological consequence. Nevertheless, multiomics associations should be regarded as hypothesis‐generating until supported by temporally resolved measurements and source‐ or target‐specific perturbation.

Figure 4 illustrates a longitudinal multiomics workflow for reconstructing interorgan regulatory networks. The workflow prioritizes candidate signaling mediators and therapeutic targets, while causal interpretation still requires route‐specific perturbation and functional validation. When combined with longitudinal sampling and perturbation‐based validation, multiomics technologies can move interorgan research from descriptive association toward mechanism‐oriented and predictive analysis, supporting biomarker discovery, therapeutic‐target prioritization, and precision medicine [143].

**FIGURE 4:**
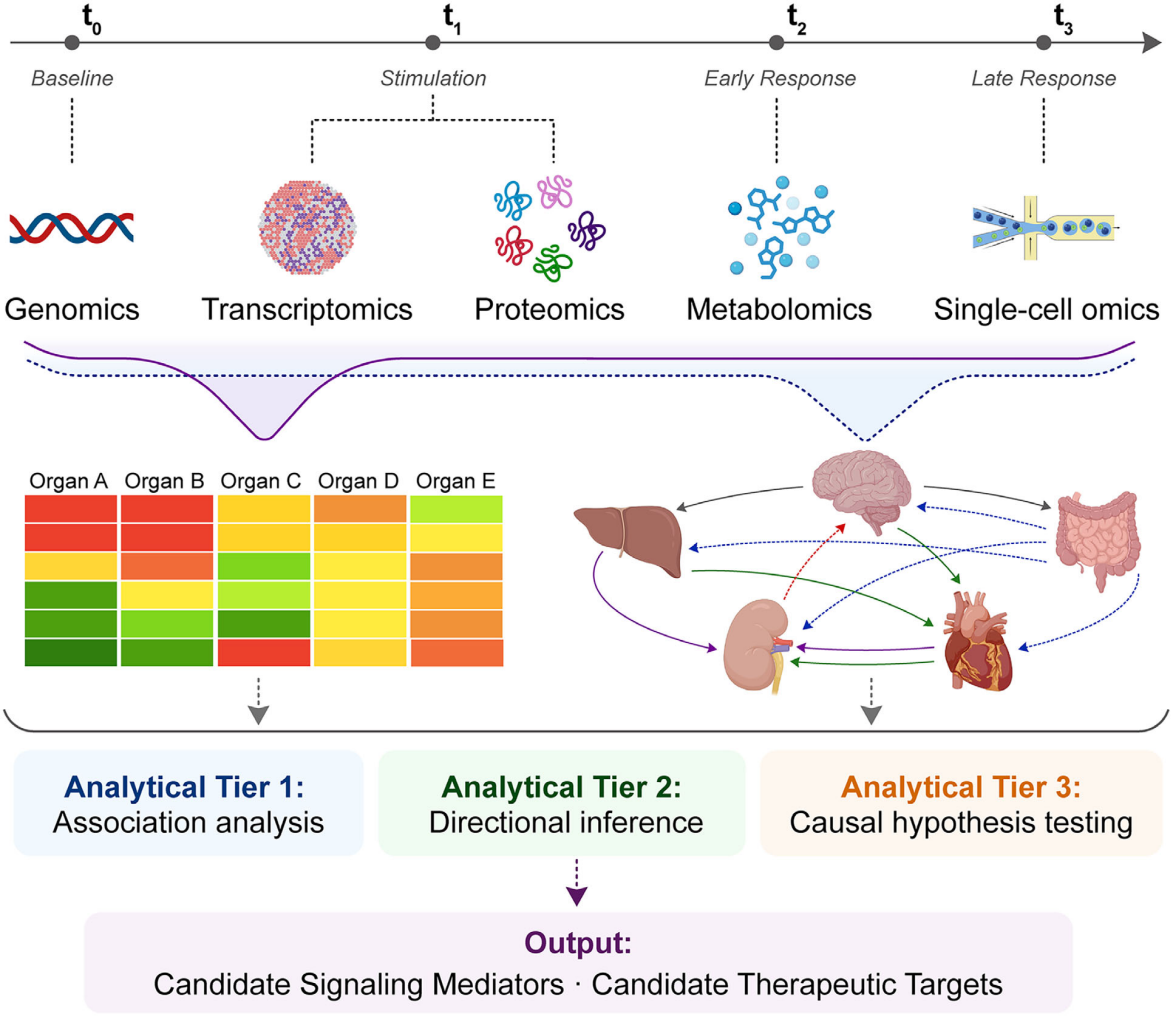
Longitudinal multiomics workflow for generating and testing interorgan network hypotheses. Molecular and cellular measurements are collected across organs and repeated time points, with genotype information used as baseline context where appropriate. Integrated data nominate associations and directional hypotheses, which require source‐ or recipient‐specific perturbation and route validation before causal interpretation. The heatmap is schematic and the outputs are candidate mediators and targets. Created with BioRender.com.

### 3.3 Computational Network Inference, Mechanistic Modeling, and Artificial Intelligence

Computational approaches in network biology range from graph‐theoretic analysis and statistical network inference to mechanistic simulation and machine learning. Classical graph‐theoretic methods characterize network structure using centrality measures, community detection, motif analysis, and path‐based network‐proximity metrics [61]. Probabilistic graphical models estimate conditional dependence structures, whereas causal graphical models seek to orient edges and estimate causal effects under explicit assumptions regarding graph structure, data‐generating distributions, confounding, and identifiability [144]. Network‐propagation methods spread prior scores from disease‐associated seed genes through molecular interaction networks to prioritize nearby candidate genes and functional modules [145]. Graph‐learning models learn representations from heterogeneous biomedical knowledge graphs containing multiple entity and relation types, thereby supporting drug‐repurposing and target‐prioritization tasks [62]. Mechanistic models, including ordinary and partial differential equation (ODE/PDE), physiologically based pharmacokinetic (PBPK), quantitative systems pharmacology (QSP), and agent‐based models, simulate dynamic responses, whereas artificial intelligence and digital twins support high‐dimensional prediction and patient‐specific hypothesis testing. Artificial intelligence also supports multiomics integration and organ‐on‐chip drug evaluation, although model‐derived candidates require experimental validation [146, 147, 148].

Computational modeling combines data‐driven inference with physics‐based simulation to quantify cross‐organ dependencies and predict system‐level responses. Dynamic multiomics modeling has identified organ‐specific metastatic states and candidate transition trajectories in lung adenocarcinoma [149]. Patient‐specific extracorporeal membrane oxygenation (ECMO) simulations integrating vascular geometry, hemodynamics, and oxygen transport have further shown how extracorporeal flow redistributes oxygen delivery between the coronary and cerebral circulations [150]. In organ‐on‐chip systems, controlled perfusion, shear stress, substrate stiffness, and tissue deformation can be combined with computational or multiphysics modeling to examine how mechanical inputs alter biochemical signaling and functional coupling across connected compartments [18]. These approaches provide complementary predictive and mechanistic frameworks, although their translational value requires external and prospective validation.

Computational models for interorgan research include mechanistic, network‐based, and data‐driven frameworks. ODE/PDE models capture continuous biochemical dynamics, whereas PBPK models quantify drug disposition across organs [65, 151]. QSP models further integrate drug action with disease pathways, while network‐physiology approaches characterize time‐varying coupling between organ systems [66, 152]. Probabilistic and causal graphical models estimate conditional or directional dependencies from multiomic and longitudinal measurements, but these relationships require perturbation‐based and functional validation before causal interpretation [153]. Machine‐learning models identify predictive patterns in high‐dimensional datasets, whereas patient‐specific digital twins integrate multiscale data for individualized simulation. Interpretability, generalizability, and prospective validation remain important constraints [146, 154, 155].

Reliable application of computational models requires appropriate calibration, independent validation, uncertainty quantification, and transparent reporting. Mechanistic and digital‐twin models should be evaluated against data not used for calibration, while uncertainty in model parameters, structure, and predictions should be explicitly characterized [156, 157]. Models intended for clinical decision support further require prospective evaluation in their intended setting, including assessment of safety, human–model interaction, and clinical utility [158].

Computational modeling and artificial intelligence enable quantitative and predictive analyses of interorgan interactions, but current models remain limited by incomplete biological knowledge, uncertain assumptions, and heterogeneous or nonrepresentative data [159]. Progress therefore depends on standardized data acquisition, explicit reporting of uncertainty, and tighter coupling between computational predictions and experimental validation. For hierarchical interorgan signaling, computational models should distinguish direct source‐to‐target effects from secondary downstream propagation and explicitly represent return signals or feedback when supported by temporal and perturbational data. Candidate hierarchical relationships involving a source organ, a primary recipient organ, and subsequent secondary recipient organs can be tested as dynamic network hypotheses rather than inferred from simultaneous correlations alone.

Figure 5 summarizes the use of artificial intelligence and computational modeling for predictive simulation of interorgan interactions, whereas Table 1 integrates the major experimental and computational approaches with their network scales, applications, and limitations. Experimental platforms and multiomics technologies provide complementary means to model cross‐organ physiology and identify candidate mediators. Computational modeling and AI integrate heterogeneous biomedical data to support predictive analysis and precision‐medicine applications [160].

**FIGURE 5:**
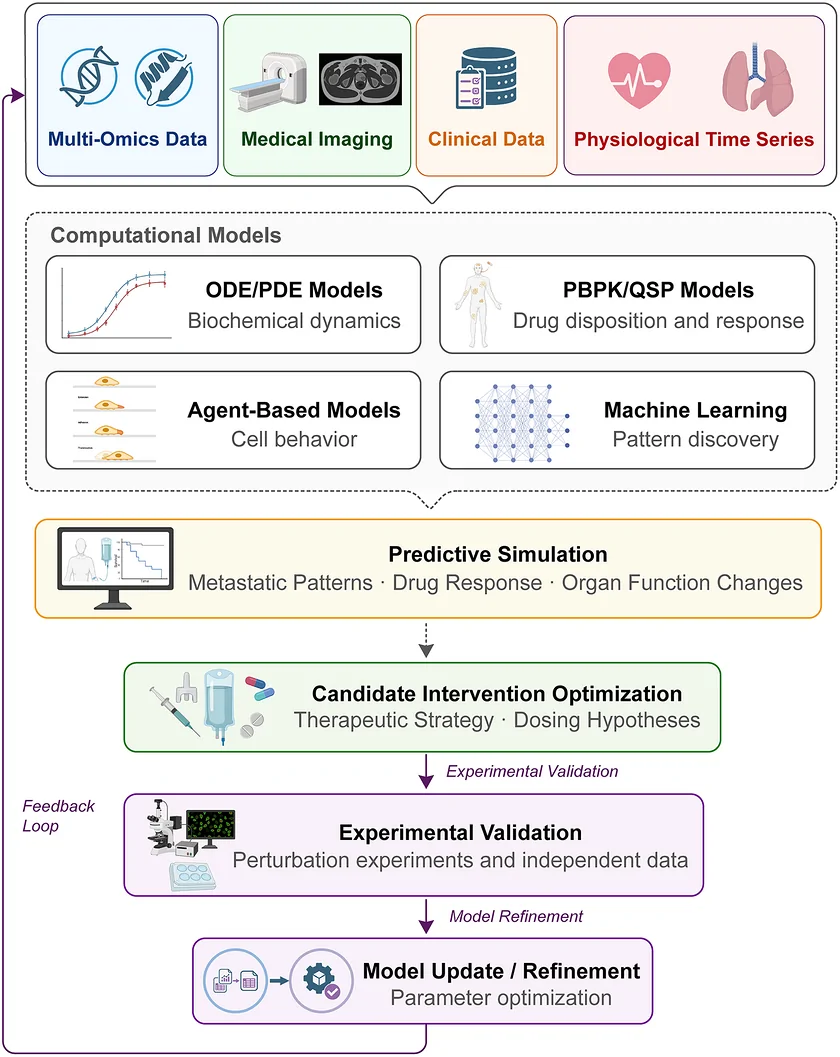
Computational modeling and experimental validation of interorgan response predictions. Multiomics, imaging, clinical, and physiological data inform differential equation, pharmacokinetic, systems pharmacology, agent‐based, and machine‐learning models. Predicted responses and candidate interventions undergo experimental testing and evaluation with independent data, followed by model refinement. Created with BioRender.com.

### 3.4 Causal Validation and Multiscale Integration

Network reconstruction should be treated as hypothesis generation rather than proof of biological causality because the same observed edge may reflect direct regulation, indirect mediation, shared upstream control, or model‐specific bias [144]. Strong causal evidence therefore requires temporally ordered perturbations that test necessity and sufficiency, together with dose–response analysis, pathway blockade, phenotypic rescue, and replication in an independent model [69, 114]. For interorgan networks, this evidentiary chain must additionally identify the source tissue, the transmitted mediator or migrating cell, the transport or transmission route, the relevant recipient population, and the functional consequence in the target organ. For hierarchical multiorgan cascades, this evidentiary chain should be established at successive stages to distinguish the initiating source organ, the primary recipient organ, secondary downstream organs, and feedback signals returning to upstream nodes. Temporal precedence alone is insufficient. Stage‐specific or route‐specific perturbation is required to determine whether an intermediate organ actively transmits the signal or merely responds in parallel to a shared upstream driver. Studies of the gut–liver axis illustrate how source manipulation, route‐specific intervention, target‐cell analysis, and rescue can convert a cross‐organ association into a testable mechanism [161].

Validation strategies should be matched to the communication modality. Neural circuits require anatomical tracing combined with selective activation or inhibition [162], whereas humoral pathways benefit from cross‐circulation or parabiosis and direct replacement or depletion of candidate mediators [163]. No single platform can satisfy all causal criteria, so claims should be triangulated across whole‐animal models, human organoids, and organ‐on‐chip systems, with each platform used at the scale for which it is most informative [164, 165, 166]. Human‐derived organoids and microphysiological systems complement animal studies by providing tighter control over cell identity, exposure, transport routes, and species‐specific responses while preserving selected tissue or organ interactions [167, 168]. Independent replication, prespecified performance criteria, and benchmarking against accepted comparators are required before a mechanistic or predictive signal can be considered robust.

Multiscale models should define their context of use, scale‐bridging assumptions, and the spatial and temporal scope of prediction [169]. Hybrid approaches can combine mechanistic models with data‐driven learning, but dynamical models require structural and practical identifiability analysis before fitted parameters are interpreted or predictions are propagated across biological scales [58]. Machine‐learning and digital‐twin components should transparently report data provenance, missing‐data handling, calibration, subgroup performance, model updating, and intended use [170]. Their predictions should remain investigational until independently benchmarked and validated in the intended population. Perturbation‐based causal evidence and scale‐explicit modeling connect descriptive network maps with testable biomedical applications.

No single technology can resolve a multiscale biological network. Robust reconstruction therefore depends on combining high‐dimensional molecular profiling with human‐relevant engineered systems that preserve relevant transport and tissue context. Automated imaging and machine‐learning analysis can increase throughput and detect complex phenotypes, but their outputs are most informative when linked to interpretable biological readouts rather than treated as self‐sufficient predictions [171]. Mechanistic confidence increases when candidate edges are challenged through controlled changes in flow, mechanical cues, or organ coupling and when the resulting phenotype is reproduced or rescued in an independent platform [172]. These considerations support an iterative framework of discovery, perturbation, validation, and model refinement, with the selected platform matched to the biological scale and intended translational claim.

## 4 Biomedical Applications of Network Biology

Network biology organizes disease‐associated components into interpretable modules for biomarker discovery, therapeutic‐target prioritization, and treatment‐response prediction [173]. Human‐relevant organoid and organ‐on‐chip platforms enable experimental testing of network‐derived hypotheses across cellular, tissue, and physiological scales [174]. These applications span molecular, intracellular, single‐cell, multicellular, microbial, disease, and interorgan networks, with the examples below emphasizing interorgan biology within the broader framework of network medicine and precision healthcare [175]. To distinguish translational maturity, a descriptive, claim‐specific framework is used in Sections 2.6 and 4 and Table 2. Level 1 denotes mechanistic or technical proof of concept, including in vitro, ex vivo, computational, invertebrate, and exploratory in vivo studies. Level 2 denotes disease‐relevant preclinical validation in mammalian models. Level 3 denotes observational or exploratory interventional human evidence without established clinical utility. Level 4 denotes prospective validation for a defined clinical use, including reproducibility, incremental value, clinical utility, and safety. These levels do not rank intrinsic study quality or substitute for assessment of bias and mechanistic confidence.

### 4.1 Disease Modules and Biomarker Discovery

Disease modules integrate genetic variants, regulatory circuits, protein interactions, cell states, and microbial features into network signatures that may improve disease classification. Network proximity, module activity, and single‐cell or spatial analyses can reveal shared mechanisms and the cellular sources of candidate biomarkers, whereas interorgan imaging and cross‐tissue molecular profiling extend this framework to organism‐level disease patterns. These approaches may identify subclinical cross‐organ alterations relevant to diagnosis and risk stratification, but their clinical utility requires independent prospective validation, defined sensitivity and specificity, and demonstrated incremental value over established clinical models.

Multiorgan imaging may provide complementary information on cross‐organ disease patterns by measuring coordinated phenotypes across organ systems. In a large‐scale study involving 30,444 participants, quantitative magnetic resonance imaging identified associations among heart, brain, and liver phenotypes. Increased hepatic fat accumulation, reflected by elevated proton density fat fraction, was associated with cardiac dysfunction and remodeling, which in turn correlated with cerebral white matter injury. As Level 3 observational human evidence, these findings suggest that integrated multiorgan imaging may reveal candidate cross‐organ signatures for earlier disease detection. However, independent prospective validation is required to confirm their diagnostic utility [82]. In mouse experiments, microbial depletion increased cFos expression in gut‐associated sympathetic ganglia, whereas colonization with short‐chain fatty acid‐producing bacteria suppressed this response [176]. These findings provide mechanistic evidence for microbiota‐dependent neural regulation. Their use as a clinically accessible biomarker of gut–brain dysfunction has not been established.

Beyond organ‐level signals, the identification of cross‐tissue multicellular coordination modules provides an additional layer for detecting aberrant interorgan interactions. By constructing a comprehensive single‐cell transcriptomic atlas encompassing 35 human tissues and over 2.29 million cells, researchers used the CoVarNet framework to identify 12 cross‐tissue CMs that capture coordinated cellular variation across tissues. In healthy states, tissue‐specific CMs are relatively stable, whereas their activity is altered in cancer, and CM activity and associated marker combinations may therefore support early cancer detection and disease‐progression monitoring, although prospective validation is required. As exploratory Level 3 human evidence, this approach provides preliminary support for diagnostics based on disrupted interorgan interaction networks [59]. Independent validation in prospective cohorts, defined sensitivity and specificity, and evidence of incremental value over existing clinical models are required before any interorgan interaction‐based diagnostic approaches can be considered clinically actionable [177].

### 4.2 Network‐Guided Therapeutic‐Target Identification

Network‐guided therapy aims to modulate disease modules rather than isolated targets through hub or bottleneck prioritization, network‐based drug repositioning, and multitarget intervention across compensatory pathways. Therapeutic prioritization should account for tissue specificity, redundancy, and off‐target network disruption, particularly when highly connected nodes also support normal physiology. Interorgan axes extend this concept because soluble mediators, EVs, neural transmission, and migrating immune cells can convey pathological and therapeutic effects across distant organs. Representative mechanisms include the systemic brain–muscle IL‐6/JAK–STAT axis activated by infection or chronic disease and microbiota–gut–brain circuits mediated by microbial metabolites and neural signaling [176, 178]. Such mechanisms identify cross‐organ signaling nodes as therapeutic candidates, although efficacy and safety remain context dependent.

Mechanistic studies of interorgan interaction networks have identified candidate precision interventions. The gut–joint mechanism identifies intestinal FXR and GLP‐1 signaling as preclinical intervention points, although efficacy in human osteoarthritis remains unvalidated [78]. In infection and chronic disease models, central nervous system‐derived reactive oxygen species induce interleukin‐6 (IL‐6) expression, activate the JAK–STAT pathway in skeletal muscle, and impair motor function. These Level 2 data support targeting IL‐6 signaling or JAK activity as candidate strategies for alleviating muscle dysfunction [178]. Following cardiac arrest/cardiopulmonary resuscitation, Enterobacteriaceae expansion and lipopolysaccharide release activate macrophage TLR4 signaling, upregulate triggering receptor expressed on myeloid cells 1 (TREM1), and promote the migration of intestinal macrophages to the brain. Level 2 preclinical data indicate that TREM1 inhibition, microbiota modulation, or blockade of the TLR4–NF‐κB/AP‐1 pathway can mitigate secondary brain and intestinal injury [179].

In a mouse model of poststroke depression, Xiaoyaosan modulated gut microbial metabolism and P2 × 7R/NLRP3 inflammasome signaling and alleviated depression‐like behavior, providing Level 2 preclinical evidence for a network‐guided therapeutic hypothesis [180]. In autism spectrum disorder models, transplantation of patient‐derived gut microbiota induces ASD‐like behaviors partly through reduced taurine and 5‐aminovaleric acid levels, whereas restoring these metabolites or modifying microbial pathways improves behavioral abnormalities, providing Level 2 support for microbiota‐informed therapy [181]. Chronic social defeat stress similarly reduces *Lactobacillus* abundance and promotes the differentiation and meningeal migration of IL‐17‐producing γδ17 cells. Level 2 preclinical data show that *Lactobacillus* supplementation, Dectin‐1 inhibition, or peripheral γδ T‐cell depletion alleviates stress‐induced social avoidance [182].

Human intervention studies also provide evidence of cardiovascular responses to microbiota‐directed treatment. In a randomized Phase II trial in untreated essential hypertension, supplementation with acetylated and butyrylated high‐amylose maize starch reduced 24‐h systolic blood pressure [183]. This constitutes exploratory human interventional evidence at Level 3, without establishing a complete interorgan mechanism or validated routine clinical use.

### 4.3 Drug–Response Prediction and Personalized Network Medicine

Patient‐specific network medicine integrates molecular profiles, cell‐state composition, microbial features, organ function, treatment exposure, and longitudinal clinical data to predict heterogeneous responses. Organoids, organ‐on‐chip systems, computational models, and digital twins can test how interventions propagate through connected pathways and organ systems. These platforms currently function primarily as proof‐of‐concept or preclinical tools and require prospective benchmarking before they can guide routine clinical decisions.

Level 1 technical proof‐of‐concept studies show that fluidically coupled multiorgan microphysiological systems can reveal how drug metabolism in one tissue alters efficacy or toxicity in another [184]. In a vascularly linked heart–liver–bone–skin chip, liver metabolism of doxorubicin was integrated with pharmacokinetic–pharmacodynamic and cardiac toxicity readouts, demonstrating information unavailable from isolated tissues [71]. Patient‐derived iPSC‐based organ‐on‐chip models offer a route to individualized disease and pharmacogenomic testing, but routine patient‐specific deployment remains unvalidated [185]. Level 1 disease‐model proof‐of‐concept evidence from a gut–liver–adipose chip further supports disease‐relevant modeling of metabolic crosstalk in nonalcoholic fatty liver disease [72]. However, routine personalized application remains limited by patient‐specific cell sourcing and maturation, platform complexity, scalability, standardization, clinical benchmarking, and evolving regulatory acceptance [186].

In gastric cancer, patient‐derived organoids from a living biobank were separately cocultured with matched cancer‐associated fibroblasts or T cells to model tumor–stromal and tumor–immune interactions [187]. Although these models do not constitute a multiorgan system, genomic profiling and drug‐sensitivity testing showed high concordance with the source tumors and exploratory agreement with patient responses, providing Level 3 evidence for personalized drug screening.

Current interorgan models can support experimental evaluation of cross‐organ pharmacokinetics, pharmacodynamics, efficacy, and toxicity, and may help prioritize patient‐specific therapeutic hypotheses. However, the available evidence remains predominantly at the proof‐of‐concept or preclinical stage. The use of these platforms for individualized target selection, dose optimization, or clinical treatment decisions will require prospective benchmarking against patient outcomes, standardized performance criteria, and regulatory validation.

### 4.4 Engineered Network Models, Digital Twins, and Regenerative Applications

Engineering approaches create controllable biological‐network models by organizing multiple cell types, tissue interfaces, vascular channels, mechanical cues, and circulating signals within defined architectures. These platforms enable studies of multicellular communication, tissue‐interface regulation, drug transport, and regenerative responses, whereas digital twins integrate patient‐specific measurements with mechanistic and data‐driven simulations. Because the examples below are mainly multitissue or tissue‐interface models rather than true interorgan systems, they are included as representative applications of engineered network biology [188]. Filament‐diameter‐adjustable 3D printing enables continuous control of gradient pore architecture, providing a scalable strategy for constructing heterogeneous biomimetic scaffolds [189]. Mineralized collagen and in situ mineralization strategies reproduce selected compositional and microarchitectural features of bone [190, 191]. Continuous manufacturing of integrated bone‐periosteum scaffolds further improves structural integration and supports bone regeneration [192]. Collagen‐based bioinks provide extracellular‐matrix environments for regenerative constructs [193]. These material strategies control candidate network inputs such as architecture and matrix composition, but they demonstrate engineered network function only when linked to measured interactions among cells or tissue compartments.

Level 1 in vitro studies have demonstrated controlled gut–liver microphysiological coupling [172]. Cardiovascular tissue‐engineering platforms also support the integration of myocardial, vascular, and microcirculatory functions [194]. Level 2 preclinical studies further show that biomimetic constructs can promote tendon–bone interface repair and uterine reconstruction by reproducing spatially organized tissue cues and extracellular‐matrix microenvironments [195, 196].

Patient‐derived models may support personalized drug–response modeling, whereas biomimetic materials provide controllable platforms for preclinical evaluation of tissue‐interface repair [197]. These applications remain predominantly at the proof of concept or preclinical stage and require further biological, technical, clinical, and regulatory validation. Figure 6 summarizes investigational biomedical applications of network biology, including candidate biomarker discovery, mechanism‐guided therapeutic investigation, experimental drug–response modeling, and regenerative multitissue engineering.

**FIGURE 6:**
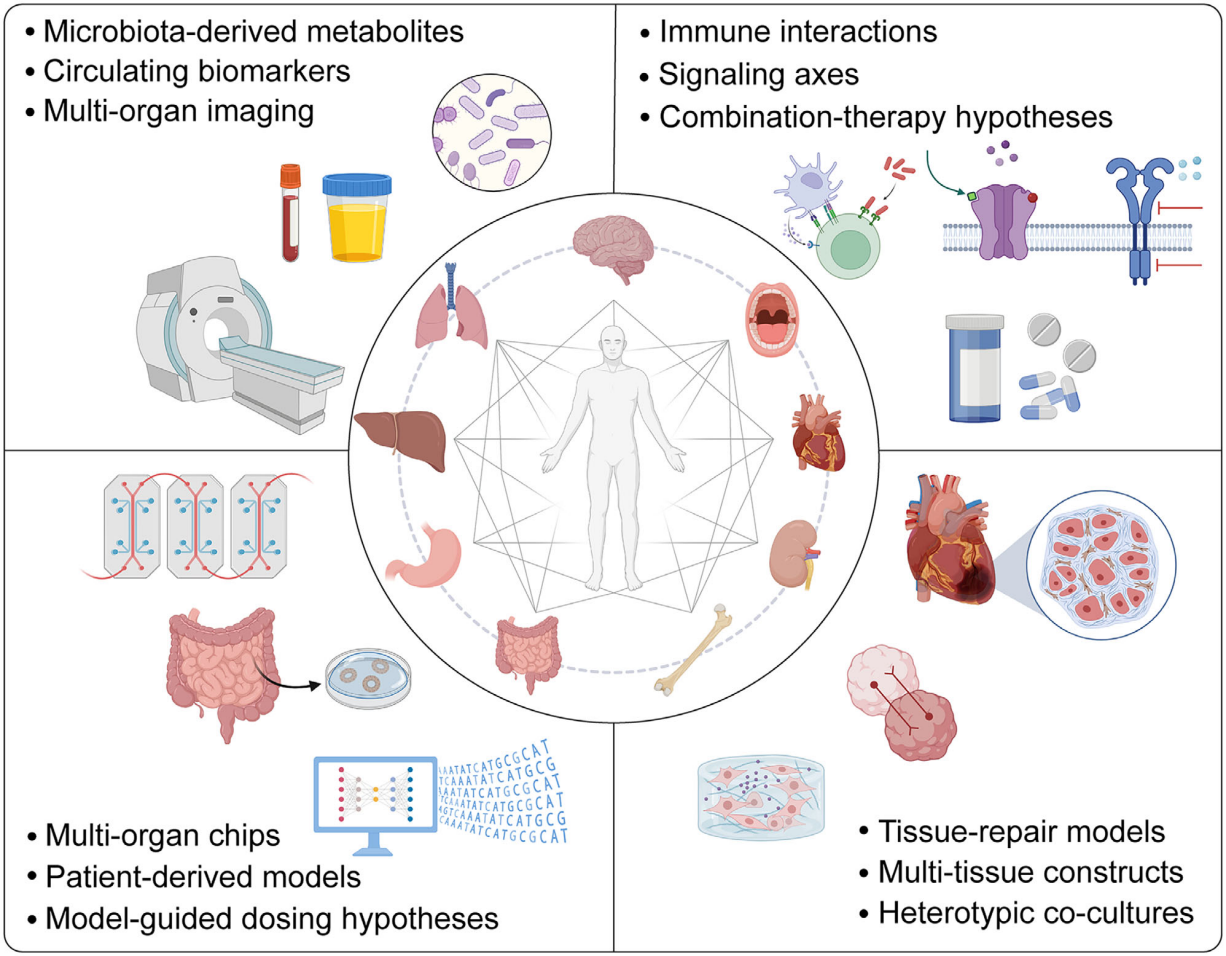
Investigational biomedical applications of network biology. Network biology supports emerging applications in biomarker discovery, mechanism‐guided therapeutic investigation, experimental drug–response modeling, and regenerative multitissue engineering by integrating microbial, circulating, imaging, patient‐derived, organ‐on‐chip, and computational data. Most representative applications remain at L1–L3 translational‐maturity levels and require further validation before use in a defined clinical setting. Created with BioRender.com.

Table 2 links the independent mechanistic properties introduced in Section 2.6 to representative preclinical, human observational, and experimental‐platform evidence discussed here. Its shared study‐level entries allow comparison of biological mechanism and translational maturity without assigning evidence levels to communication categories. However, the supporting evidence remains concentrated predominantly at Levels 1–3, encompassing mechanistic or technical proof‐of‐concept studies, preclinical validation, and observational human associations. These applications should therefore be regarded as emerging research directions rather than established diagnostic tools, validated precision therapies, or clinically qualified decision‐support platforms. Translation into routine practice will require independent prospective validation, standardized performance metrics, benchmarking against current clinical approaches, demonstration of clinical utility, and regulatory evaluation.

## 5 Major Challenges in Network Biology

Despite rapid progress, network biology remains constrained by incomplete and context‐dependent interaction maps, heterogeneous data quality, limited temporal resolution, uncertain causal directionality, and difficulties in integrating networks across biological scales [115]. Predictive models face additional challenges involving interpretability, empirical validation, standardization, and clinical translation [198]. These challenges affect every branch of the field, although their relative importance differs across molecular, cellular, microbial, tissue, organ, and patient‐specific networks. These limitations can be organized into methodological, mechanistic, computational, translational, and governance domains, with interorgan systems providing recurring examples of multiscale complexity [199].

### 5.1 Data Quality, Standardization, and Reproducibility

Network estimates are highly sensitive to sampling depth, preprocessing and normalization choices, feature selection, and batch effects. Cell‐type annotation quality, sparse measurements, and the absence of experimentally established ground‐truth edges can further affect downstream network reconstruction and evaluation [200]. Reproducible network biology therefore requires standardized performance metrics, well‐characterized reference datasets, transparent analytical workflows, and evaluation against independent or real‐world benchmarks. Single‐cell studies should explicitly evaluate sparsity, batch correction, and the stability of biological conclusions across integration and feature‐selection strategies. Microbiome studies should account for compositional structure, confounding, repeated measurements, and method‐dependent variation in differential‐abundance results [201, 202]. Engineered tissue platforms should use harmonized quality‐control criteria and cross‐site benchmarking to distinguish reproducible biological properties from platform‐dependent variability.

A practical standardization framework should encompass three interdependent levels covering measurement procedures, computational analysis, and model‐system performance [203]. At the measurement level, minimum metadata should document tissue procurement, processing intervals, perturbation dose and timing, sequencing depth, sensor calibration, missingness, and predefined sample‐exclusion criteria [204]. At the computational level, studies should report preprocessing decisions, network‐inference parameters, negative controls, confidence intervals, software versions, and accessible analysis code. Major modules and edges should also be tested for stability across reasonable preprocessing choices, alternative feature‐selection strategies, and independent analytical pipelines. At the model‐system level, organoids and microphysiological platforms require quantitative benchmarks for cell identity, maturation, tissue scaling, flow conditions, medium composition, barrier integrity, and baseline function, together with interlaboratory comparison against relevant human reference data [29].

Reproducibility should also be evaluated at the level of the scientific claim rather than only at the level of the dataset. An inferred network edge should receive graded confidence based on orthogonal measurement, replication in an independent dataset, robustness to alternative analytical methods, and consistency with perturbation data. Uncertainty should be reported for nodes, edges, modules, and model outputs because visually complete network diagrams can otherwise obscure incomplete observability, uncertain edge structure, and model‐form uncertainty.

### 5.2 Mechanistic Incompleteness and Organ‐Specific Targeting

Key challenges in elucidating interorgan interaction mechanisms lie in the incomplete identification of the signal carriers, molecular targets, and upstream–downstream regulatory networks that mediate cross‐organ communication. For example, in the kidney–brain–heart axis, viral tracing and optogenetic approaches have delineated a multisynaptic neural pathway involving renal afferent nerves, the subfornical organ (SFO), paraventricular nucleus (PVN), and RVLM. However, the critical mechanisms by which renal injury‐derived signals are sensed and encoded by renal afferent nerves remain largely unresolved [205]. Similarly, in liver–kidney communication studies, metabolically labeled liver‐derived RNA has been detected in the kidney, supporting potential interorgan RNA transfer. Yet, although SLAM‐seq confirms the presence of RNAs across organs, it cannot define their transport vehicles, such as exosomes, lipoproteins, or free RNA, or resolve their downstream targets and functional networks in the kidney, leaving key mechanisms of RNA‐mediated liver–kidney communication unresolved [206].

Clinical observations in chronic thromboembolic pulmonary hypertension (CTEPH) illustrate the need to relate hemodynamic recovery to molecular remodeling. Human myocardial biopsy analyses before and 12 months after pulmonary endarterectomy (PEA) showed reduced hypertrophy and fibrosis, although residual septal fibrosis indicated incomplete reverse remodeling [207]. These findings support investigation of complementary targets for right ventricular recovery after hemodynamic correction. A related challenge is that many crosstalk mediators exert physiological functions in multiple organs, making organ‐specific targeting difficult and raising safety concerns. For instance, pigment epithelium‐derived factor (PEDF), a key mediator of the liver–gut axis that maintains ISC homeostasis by suppressing Wnt/β‐catenin signaling, also plays essential roles in hepatic metabolism and retinal neuroprotection. Consequently, therapeutic targeting of PEDF for intestinal disorders may inadvertently disrupt functions in nontarget organs [161].

Even interventions directed at a single organ can destabilize systemic homeostasis when their downstream effects propagate through cross‐organ signaling networks, emphasizing the need to evaluate both target‐organ efficacy and nontarget‐organ consequences. Available delivery strategies still provide incomplete control over tissue selectivity, exposure duration, and nontarget‐organ accumulation, limiting the translation of interventions directed at interorgan signaling networks. Targeted nanoparticle and localized biomaterial delivery systems, including magnesium‐containing microspheres and intra‐articular carriers, illustrate how spatially restricted immunomodulation and drug release may reduce systemic exposure and off‐target network disruption [208, 209, 210].

### 5.3 Network Dynamics, Directionality, and Causality

Most biological networks are dynamic, state dependent, and adaptive, with regulatory connections being rewired across developmental, physiological, and pathological states [211]. Static measurements can miss transient edges, feedback, compensation, and temporal ordering, while observational associations cannot reliably distinguish direct interactions from shared upstream regulation or other confounding processes [212]. Progress will therefore depend on longitudinal multiomics profiling and continuous or high‐frequency physiological sensing that capture changes across relevant temporal windows [213]. Controlled perturbations, rescue experiments, and causal models evaluated against independent interventional datasets are additionally required to move from association toward mechanism. Interorgan studies are especially demanding because signal production, systemic transport, tissue uptake, neural transmission, immune‐cell migration, and downstream responses may occur over markedly different timescales [111].

Directionality and causality should be treated as distinct evidentiary stages [214]. Temporal precedence or directed graphical models can prioritize candidate upstream relationships, but their conclusions remain assumption dependent and may be distorted by unmeasured confounding, unequal sampling intervals, feedback, and model misspecification. For interorgan axes, a causal claim should ideally establish an evidence chain comprising source‐organ perturbation, identification of the transmitted mediator or migrating cell, route‐specific transport, engagement of a defined recipient population, a measurable target‐organ response, and rescue or reversal of the phenotype.

Experimental design must also match the characteristic timescale of each network edge. Neural transmission can occur within milliseconds, whereas changes in secretion, immune‐cell recruitment, and tissue remodeling may develop over longer and partly overlapping intervals. Sampling should be matched to the measured endpoint and experimental context. Multirate sampling, synchronized physiological recording, repeated omics measurements, and temporally controlled perturbations are therefore preferable to a single uniform sampling schedule. Compensatory pathways should be tested explicitly through sequential or combinatorial perturbations, because the apparent absence of an effect after blocking one route may reflect functional buffering or network redundancy rather than lack of biological importance [215].

### 5.4 Multiscale Integration and Model Interpretability

Integrating networks across molecular, cellular, microbial, tissue, and organ scales requires explicit rules for translating local interactions into higher order phenotypes. Differences in spatial resolution, temporal dynamics, edge definitions, and biological context can create substantial representation gaps between adjacent scales. Parameter nonidentifiability, incomplete mechanistic knowledge, and reliance on black‐box prediction can further limit biological interpretation and translational reliability. Hybrid mechanistic‐data‐driven models, uncertainty quantification, interpretable graph learning, and perturbation‐based validation are required to identify reproducibly predictive and potentially actionable network features.

Cross‐scale integration is most interpretable when models define explicit interface variables that connect adjacent levels. Such variables may include ligand concentration and receptor occupancy between molecular and cellular scales, cell‐state composition and secretory flux between cellular and tissue scales, or blood flow, neural activity, and immune‐cell migration between organ modules [59]. Hierarchical and modular models can then be calibrated and validated locally before being assembled into a network of networks, thereby reducing the risk that uncertainty or model error at one scale is silently propagated through the entire system.

Interpretability should extend beyond post hoc feature ranking and should identify biologically meaningful regulatory modules, paths, and intermediate variables that contribute to a prediction. A useful explanation should clarify which assumptions, parameters, measurements, and model structures drive the result, whether alternative plausible models yield the same conclusion, and how uncertainty affects the recommended action [157]. Hybrid models that constrain machine learning using mass balance, pharmacokinetics, signaling logic, anatomical structure, or other established biological knowledge may improve mechanistic plausibility and reduce the effective hypothesis space. Nevertheless, such models still require sensitivity analysis, external and distribution‐shift testing, and validation against independent perturbations to demonstrate that an explanation is mechanistically meaningful rather than merely statistically convenient [216].

### 5.5 Clinical Translation, Ethics, and Governance

Clinical network models must demonstrate analytical validity, biological validity, reproducibility, incremental predictive value, and clinically meaningful utility [217]. Patient‐specific networks and digital twins also raise concerns regarding data provenance, privacy, algorithmic bias, version control, accountability, and regulatory oversight [218]. Network‐derived interventions should be evaluated for unintended effects on connected pathways and nontarget organs, particularly when hubs or broadly shared modules are targeted.

Translation should begin with a predefined intended use, target population, decision context, comparator, and clinically meaningful endpoint. Prospective evaluation should assess calibration, discrimination, subgroup performance, robustness across sites and instruments, effects on clinical workflow and clinician behavior, and incremental value beyond established clinical variables [219]. Version locking, predefined change‐control procedures, and postdeployment surveillance are particularly important for adaptive systems because performance can drift as patient populations, measurement technologies, and clinical workflows change.

Governance should address data provenance, consent, privacy, representativeness, version control, responsibility for model‐supported decisions, and procedures for investigating failure [220]. Patient‐specific networks and digital twins should function as transparent decision‐support tools rather than autonomous substitutes for clinical judgment. Because interventions directed at hubs or shared pathways may redistribute risk across connected organs, organ‐specific delivery, cross‐organ safety monitoring, and stopping criteria should be incorporated into development from the outset [221].

These challenges form an evidence chain in which measurement bias distorts network reconstruction, weakens causal interpretation, and propagates into multiscale predictions and clinical decisions. Claim strength should therefore match the maturity of the supporting evidence. Descriptive claims require reproducible measurements, causal claims require temporally resolved perturbation and functional rescue, predictive claims require external validation and uncertainty assessment, and clinical claims require prospective demonstration of utility and safety. Models should prioritize context‐specific networks that explain defined phenotypes and remain generalizable across relevant populations and conditions [222].

A staged validation workflow should define the signal source, carrier, route, recipient, and system‐level consequence. Longitudinal or spatial profiling should then be combined with targeted perturbation and rescue, followed by testing of supported networks in independent and human‐relevant systems. Translation should also assess compensatory pathways, nontarget‐organ effects, and long‐term network reorganization. Near‐term priorities include multicenter reference datasets, standardized data‐quality and reproducibility benchmarks, perturbation‐resolved human‐relevant models, and prospective evaluation of patient‐specific predictions.

## 6 Future Prospects

Future progress in network biology will depend on combining standardized human‐relevant experimental systems with longitudinal multiomics approaches capable of resolving dynamic biological states [223]. Spatially resolved profiling and artificial intelligence can provide tissue context and support the integration of molecular and cellular information across organs and biological scales. Mechanistic models and patient‐specific digital twins should undergo rigorous verification, uncertainty quantification, and prospective clinical validation before being applied to clinical decision support. The following subsections outline technological and conceptual directions for moving from descriptive network mapping toward predictive, patient‐specific, and intervention‐oriented models, with interorgan interaction networks serving as an organism‐level test bed for multiscale integration.

### 6.1 Convergent Experimental and Computational Platforms

Progress in interorgan research increasingly depends on integrating experimental biology, bioengineering, multiomics, and computational modeling. Linking human‐relevant experimental systems with multiscale and patient‐specific computational models may improve mechanistic understanding and facilitate the development of precision‐medicine strategies [224]. A key next step is to establish closed experimental–computational loops in which model predictions guide targeted perturbations and the resulting experimental data iteratively refine model structure and parameters.

AI‐assisted approaches are expanding the analysis and predictive use of organoid and bioengineered systems. In brain organoids, high‐throughput imaging has supported neurotoxicity prediction, while multiomics and imaging integration can reveal nonlinear relationships across molecular and cellular layers [225, 226]. When extended across tissues, these methods may facilitate reconstruction and prediction of cross‐organ responses [227]. The 3DCellScope platform quantitatively characterizes nuclear, cellular, and regional architecture in pancreatic ductal adenocarcinoma organoids [228], whereas DILITracer uses bright‐field liver‐organoid images to predict drug‐induced hepatotoxicity [171]. Model‐driven vascular design integrating vascular‐tree generation, fluid dynamics, and 3D bioprinting has also accelerated organ‐scale network generation and improved cell viability in perfused constructs [229].

Big‐data approaches provide a complementary framework for studying organism‐level relationships. Multidimensional data from more than 10 million individuals across six population‐based cohorts were used to train large language models (LLMs) for estimating systemic and organ‐specific aging, revealing associations between aging discordance and disease risk [230]. Externally controlled perturbation platforms may eventually enable spatially restricted testing of network‐derived therapeutic targets, although their application to interorgan networks remains unproven [231].

### 6.2 Spatiotemporal Mapping and Dynamic Network Reconstruction

Spatiotemporal resolution is essential for distinguishing synchronous organ responses from ordered signal propagation, delayed adaptation, and feedback across organs. In one study, 6‐month‐old Fischer 344 rats underwent 8 weeks of endurance training combined with multitissue, multiomics profiling to decode exercise‐induced interorgan interactions across space and time. Spatially, prolonged training led to convergent, sex‐independent molecular responses in specific skeletal muscles and the heart, accompanied by coordinated changes in blood, liver, and small intestine, forming an integrated circulatory–metabolic–muscle network. Temporally, organs including the small intestine, adipose tissue, and adrenal gland showed distinct response profiles across training phases, revealing coordinated temporal remodeling without by itself establishing directed signal transfer between organs [20].

These longitudinal observations indicate that the biological meaning of an interorgan edge depends not only on its participating organs and mediators but also on when the interaction emerges, how long it persists, and whether its direction changes with physiological or pathological state [232]. Near‐term work should test whether spatiotemporal mapping of neural circuits and multiorgan responses during exercise or stress can yield candidate biomarkers of organ crosstalk. A complementary priority is to determine whether longitudinal multitissue multiomics, combined with dynamic modeling and causal‐inference methods, can recover the temporal ordering of cross‐organ signaling events and prioritize directional relationships for perturbational validation. Addressing these questions would move spatiotemporal mapping beyond descriptive profiling toward perturbation‐guided reconstruction of directional interorgan networks.

### 6.3 Extreme‐Environment Adaptation as a Systems‐Level Model

Extreme‐environment exposure disrupts coordinated physiology across organ systems and can reveal network states associated with adaptation or failure [233]. At high altitude, longitudinal human metagenomic studies show rapid gut–microbiota remodeling, with Blautia A species associated with improved intestinal barrier function, reduced inflammation, and hypoxia acclimatization [234]. Human and animal studies further link hypoxia‐induced dysbiosis and barrier dysfunction to cognitive impairment, supporting a candidate microbiota–gut–brain–immune network [235]. Acute heat stress activates the conserved HSP70‐mediated proteostatic response, whereas heatstroke causes coordinated multiorgan injury when compensatory capacity is exceeded [236].

High‐altitude acclimatization similarly requires integrated ventilatory, pulmonary vascular, cardiovascular, cerebral, renal, hematological, and metabolic responses that maintain systemic oxygen transport [237]. Cardiorespiratory adjustments, including changes in heart rate, cardiac output, pulmonary vascular tone, and cerebral blood flow, help sustain oxygen delivery during hypoxic exposure [238]. Human high‐altitude adaptation also involves gene‐regulatory variation, as long‐read sequencing identified a Tibetan‐enriched deletion that disrupts an EPAS1‐associated superenhancer and downregulates EPAS1, providing direct evidence that structural variation can rewire hypoxia‐response regulation during adaptation [239]. Extreme‐environment studies therefore provide a useful systems‐level model of adaptation, but extrapolation to chronic disease or therapeutic intervention requires direct causal and prospective validation. Future integration of longitudinal multiomics, physiological monitoring, and multiscale digital models may support the prediction of cross‐organ adaptation and failure, although these applications remain developmental and require validation and uncertainty quantification [240].

### 6.4 Predictive Biomarkers and Intervention‐Oriented Network Biology

Predictive and intervention‐oriented network biology should translate organ‐resolved measurements into prespecified, clinically testable signatures rather than treating complex network maps as biomarkers by default. Longitudinal and spatial multiomics can resolve cross‐tissue CMs and circulating signals of defined tissue origin, while human‐relevant experimental systems and patient‐specific computational models can generate experimentally testable perturbation hypotheses [59]. A candidate signature should be considered predictive only when it is measured before treatment and shown to identify differential treatment benefit rather than prognosis alone [241]. It should also be externally reproduced, calibrated in the intended population, and shown to add predictive or decision‐making value beyond established clinical variables. Recent spatial and multiomics analyses in non–small‐cell lung cancer illustrate the feasibility of integrating tissue architecture, immune context, and molecular features into signatures associated with immunotherapy outcome [242]. However, these signatures require prospective evaluation before they can be considered clinically qualified predictive biomarkers. The longer term goal is to identify organ‐specific or cross‐organ adaptation signatures that support treatment selection and prioritize hypotheses concerning treatment timing, dosing, and combination strategies while remaining mechanistically interpretable and experimentally testable [243].

Beyond these specific opportunities, several fundamental problems remain unresolved across interorgan interaction research. Identifying the tissue and cell type of origin of circulating signals will require the integration of tissue‐resolved reference maps with cell‐free molecular profiling and experimental source tracing. Monitoring dynamic signaling across multiple organs and reconstructing the temporal sequence of multiorgan cascades will require longitudinal multiomics, synchronized physiological recording, and continuous or repeated organ‐resolved measurements. Compensatory pathways activated after disruption of a communication route should be investigated using sequential or higher order combinatorial perturbations rather than single‐node interventions alone [215]. Additional priorities include characterizing sex‐ and age‐dependent variation and quantitatively benchmarking species‐specific differences that may limit translation from animal models to humans [244]. Achieving organ‐specific delivery while minimizing off‐target exposure will require simultaneous evaluation of biodistribution, target‐organ activity, and nontarget‐organ accumulation [245]. Bidirectional causality should be supported by reciprocal, route‐specific perturbations and intervention‐response data rather than by correlated changes in two organs alone. Addressing these problems will require coordinated experimental, computational, and clinical efforts to move interorgan research from descriptive characterization toward predictive and intervention‐oriented network biology.

## 7 Conclusion

Network biology has evolved from the mapping of molecular interactions to a multiscale discipline that connects intracellular regulation, single‐cell states, multicellular communication, microbial ecology, disease modules, tissue organization, and interorgan physiology. This broader perspective reveals that biological function and disease emerge from coordinated interactions across nested networks rather than from isolated components. Advances in perturbation technologies, multiomics, spatial profiling, engineered models, and computational inference are progressively transforming descriptive maps into dynamic and predictive representations.

Interaction maps remain incomplete and context dependent. Inferred edges often lack causal validation, temporal and spatial measurements remain sparse, and models constructed at different scales are difficult to reconcile. Reproducibility, uncertainty quantification, interpretability, and standardized benchmarking are therefore essential. Clinical translation will additionally require prospective validation, demonstration of incremental utility, regulatory evaluation, and careful consideration of privacy and algorithmic bias.

Future progress should prioritize perturbation‐resolved single‐cell and spatial profiling, longitudinal microbial and clinical datasets, hybrid mechanistic and graph‐learning models, and experimentally testable network‐of‐networks frameworks. Patient‐specific network models and digital twins may ultimately integrate molecular, cellular, microbial, physiological, and environmental information, but their use in decision support will depend on transparent assumptions and continual validation against observed outcomes.

Within this broader landscape, interorgan interaction networks provide an important organism‐level perspective that links molecular and cellular regulation with systemic physiology. Integrating these scales within causally validated and prospectively tested models will determine whether network biology can progress from descriptive mapping to clinically informative prediction.

## Author Contributions

**Xinmeng Li**: investigation, resources, and writing – original draft. **Laizhong Zhang**: formal analysis, resources. **Yawen Ding**: data curation. **Kai Li**: validation. **Shiyuan Chang**: supervision, resources. **Linsen Pei**: data curation, resources. **Zhengwei Li**: investigation, supervision, and writing – review and editing. **Zhenzhen Chen**: funding acquisition, supervision, and writing – review and editing. All the authors have read and approved the final manuscript.

## Funding

This work was supported by grants from the National Natural Science Foundation of China (Nos. U25A2038, 82273478, 82573314), the Natural Science Foundation of Henan Province (No. 252300423868), and the Special Training Program for Clinical Medical Scientists under the “Three 100” Initiative of Henan Province (No. HNCMS202530).

## Ethics Statement

The authors have nothing to report.

## Conflicts of Interest

The authors declare no conflicts of interest.

## Data Availability

Data sharing is not applicable to this article because no new data were created or analyzed.
